# Food Insecurity, Supplemental Nutrition Assistance Program Participation and Cognitive Function Among Middle-Aged and Older Adults: Longitudinal Evidence from the Health and Retirement Study

**DOI:** 10.3390/nu18020363

**Published:** 2026-01-22

**Authors:** Ye Luo, Miao Li, Zhenmei Zhang

**Affiliations:** 1 Department of Sociology, Anthropology and Criminal Justice, Clemson University, Clemson, SC 29634, USA; miaol@clemson.edu; 2 Department of Sociology, Michigan State University, East Lansing, MI 48824, USA; zhangz12@msu.edu

**Keywords:** food insecurity, SNAP, cognition, episodic memory, mental processing, older adults, race/ethnicity

## Abstract

**Background:** Food insecurity may adversely affect cognitive function through pathways involving nutritional deficiencies, chronic stress, and comorbid health conditions, with potentially different effects across cognitive domains. Longitudinal evidence remains limited by time-varying confounding, and it is unclear whether Supplemental Food Assistance Program (SNAP) participation modifies these associations. **Objectives**: To examine the longitudinal association between food insecurity and cognitive function using marginal structural models (MSMs), and whether SNAP participation buffers these associations for overall cognition, episodic memory, and attention/mental processing. **Methods**: 30,641 adults aged ≥50 in the 1998–2020 Health and Retirement Study (HRS) contributed 156,066 person-year observations. MSMs with stabilized inverse probability of treatment weights were used to account for time-varying socioeconomic, health, and cognitive confounding affected by prior exposure. Weighted pooled linear regression models estimated marginal associations and interaction effects. **Results**: Moderate and high food insecurity were associated with lower overall cognition (b = −0.36 and −0.71, respectively; *p* < 0.001). Similar graded associations were observed for episodic memory (b = −0.22; −0.43) and attention/mental processing (b = −0.15; −0.28; all *p* < 0.001). SNAP participation significantly attenuated these associations across cognitive domains, with stronger buffering effects among non-Hispanic Black and Hispanic respondents. Effect sizes corresponded to differences equivalent to several years of cognitive aging. **Conclusions**: Food insecurity is associated with poorer cognitive function across multiple domains, while SNAP participation mitigates these associations. Despite limitations of observational data, these findings highlight the methodological value of MSMs and the potential role of food assistance programs in reducing cognitive health disparities in later life.

## 1. Introduction

Food insecurity, defined as limited or uncertain access to adequate and nutritious food [1], remains a persistent and consequential public health challenge for middle-aged and older adults in the United States. National estimates based on the U.S. Department of Agriculture (USDA)’s Household Food Security Survey Module (HFSSM) indicate that approximately one in ten adults aged 50 years and older experience food insecurity, with estimates encompassing both moderate and severe levels of food insecurity, and this figure is expected to grow as economic inequality and population aging accelerate [2,3]. The Supplemental Nutrition Assistance Program (SNAP) represents the largest federal nutrition assistance program designed to mitigate food insecurity by improving access to food resources, yet its potential role in protecting cognitive health in later life remains insufficiently understood. A growing body of literature documents strong associations of food insecurity with chronic disease, psychological distress, disability, and accelerated biological aging [4,5,6,7,8,9], suggesting multiple pathways through which food insecurity may compromise cognitive health as individuals age. Increasingly, research has linked food insecurity to poorer cognitive outcomes [10,11,12], raising concerns about the implications of food insecurity and the effectiveness of food assistance programs for maintaining cognitive health and independence among aging populations.

Although cognitive function is multidimensional, encompassing abilities such as memory, executive function, and attention, much of the existing evidence has focused on global cognitive performance, leaving less clarity regarding which specific cognitive domains are most affected by food insecurity. Indeed, several systematic reviews conclude that food insecurity is associated with poorer global cognitive performance and faster cognitive decline across the life course [10,11,12], and longitudinal studies reinforce these findings, showing that food insecurity is associated with steeper declines in overall cognition in both middle-aged and older adults [13,14,15]. However, findings are mixed across cognitive domains. For instance, two studies analyzed middle-aged and older adults from the HRS, using either a 2-item food insecurity measure or the USDA 6-Item Short Form HFSSM, found that food insecurity was significantly associated with faster memory decline [16,17] and higher dementia risk [17]. In contrast, Ludwig-Borycz et al. examined multiple cognitive domains in the HRS using the Harmonized Cognitive Assessment Protocol and reported that food insecurity measured with the USDA 6-Item Short Form HFSSM was inversely associated with executive functioning, but not with memory, language, visuospatial functioning, or orientation [18]. In addition, Kim et al. studied Medicare beneficiaries aged ≥65 years longitudinally, showing that the 5-item food insecurity measure in the National Health and Aging Trends Study predicted a faster decline in executive function, but not with declines in immediate or delayed memory recall [19].

Memory and non-memory cognitive function (attention/mental processing) represent distinct aspects of cognition shaped by different neurological systems and environmental inputs. Episodic memory, often captured by immediate and delayed recall tasks, is highly sensitive to aging, stress burden, cardiometabolic risk, and nutritional deficiencies. Non-memory cognitive function measures, such as counting tasks, vocabulary, and orientation, reflect crystallized cognitive abilities that decline more slowly and may be less responsive to short-term stressors [20]. Therefore, combining these domains into a single global cognitive score may obscure domain-specific vulnerability.

Racial and ethnic disparities add further complexity to the relationship between food insecurity and cognitive aging. Black and Hispanic older adults experience disproportionately high levels of food insecurity and face greater cumulative exposure to socioeconomic disadvantage, discrimination, and chronic stress, factors linked to accelerated cognitive decline [10,12]. Emerging evidence suggests that the cognitive consequences of food insecurity may also vary across groups. One recent study reported strong associations between food insecurity and lower cognitive performance, and persistent racial/ethnic disparities in both food insecurity and cognitive impairment [21]. However, their interaction models did not show statistically significant effect modification by race/ethnicity for incident cognitive impairment, so it remains unclear whether food insecurity exerts a greater cognitive impact among minority versus White older adults.

An equally important unresolved question is whether food assistance programs can buffer the adverse cognitive consequences of food insecurity. Existing evidence on SNAP’s role in cognitive health remains limited and inconsistent. Although participation has been linked to improvements in food access and dietary quality, studies examining the relationship between food insecurity and cognitive outcomes have reported mixed findings, with some suggesting that SNAP may mitigate nutritional risks and support cognitive health [15], while others show limited or inconsistent protective effects [13,14]. Importantly, prior research has rarely accounted for time-varying selection into food insecurity or examined whether potential buffering effects differ across cognitive domains.

The present study addresses these gaps by examining associations between food insecurity, SNAP participation, and two specific cognitive domains, episodic memory and attention/mental processing, in a large, nationally representative sample of U.S. middle-aged and older adults. Using updated analytic methods, we evaluate whether the adverse effects of food insecurity are concentrated in memory, attention/mental processing, or both; whether SNAP participation modifies these patterns; and whether associations differ across major racial/ethnic groups. Because prior research suggests that the cognitive consequences of food insecurity may differ by sex and age [10], we also conduct stratified analyses to assess the robustness of associations across sex and age groups. By focusing explicitly on domain-specific cognitive outcomes, this research provides new insight into the heterogeneous ways food insecurity may influence the aging brain and contributes to a more nuanced understanding of nutritional vulnerability, cognitive trajectories, and health equity in later life.

## 2. Materials and Methods

### 2.1. Data

This study utilized data from 1998 through 2020 of the Health and Retirement Study (HRS), conducted by the Institute for Social Research at the University of Michigan (Ann Arbor, MI, USA). The HRS is a nationally representative, longitudinal cohort study of U.S. adults aged 50 years and older, along with their spouses, every two years since its inception in 1992. To maintain sample representativeness, refresher cohorts were added every 3 waves. The study collects comprehensive information on demographic characteristics, socioeconomic status, health conditions, and economic transitions to facilitate research on aging and the determinants of health and well-being in later life [22]. The HRS protocol was approved by the University of Michigan Institutional Review Board, and written informed consent was obtained from all participants or their legally authorized representatives at each interview. For this analysis, data on food insecurity and participation in SNAP were merged from the raw HRS survey files with the RAND HRS Longitudinal File (Version 2022 V1), a harmonized dataset providing cleaned and consistently defined variables, including imputed values for income and assets. The analytic sample was restricted to respondents aged 50 years or older who were not missing on food insecurity, SNAP participation, and time-invariant covariates, not living in nursing homes, and not missing on time-variant covariates in the previous wave. After applying these inclusion criteria, the final analytic sample comprised 156,066 person-year observations from 30,641 unique respondents across the 12 survey waves. The detailed sample selection process is described in Supplementary Figure S1.

### 2.2. Measures

*Cognition*. This study assesses two subdomains of cognitive functions: episodic memory and attention/mental processing, which were included in the Telephone Interview for Cognitive Status (TICS) [23,24]. HRS assessed memory through an immediate word recall asking respondents to immediately repeat 10 words just read to them, and a delayed recall asking respondents to repeat these words a few minutes later. Episodic memory was measured by summing the scores on immediate recall and delayed recall (20 points). Attention/mental processing was assessed through serial 7 tests, which asked respondents to subtract 7 from 100 (up to five times) and backward counting for 10 continuous numbers from 20 (7 points). The overall cognitive function score was the sum of scores of episodic memory and attention/mental processing, ranging from 0 to 27, with higher scores representing better cognitive functioning. The original attention/mental processing measure in HRS also included naming questions. However, naming questions were only asked in every wave for adults aged 65 and older. Because our study included respondents aged between 50 and 64, we did not include naming questions in our cognition measures. We used the cognitive measures developed by the HRS, with missing cognition items imputed [25].

*Food insecurity*. Food insecurity was assessed with two questions that have been consistently administered in the HRS to capture economic food hardship among middle-aged and older adults, and these two questions have been used in previous studies [7,9,16]. HRS household respondents (designated as a representative spouse who answers household-level questions) were first asked “In the last 2 years, have you always had enough money to buy the food you need?” and those who answered no to this question were further asked, “In the last 12 months, did you ever eat less than you felt you should because there wasn’t enough money to buy food?” An ordinal measure of food insecurity was created based on the responses to these two questions: it was coded 0 if “yes” to the first question (low food insecurity), 1 if “no” response to both questions (moderate food insecurity), and 2 if “no” to the first question and “yes” to the second question (high food insecurity).

Although brief, this two-item measure is conceptually aligned with the USDA’s food security instruments and captures key dimensions of food access and food-related hardship. Prior research demonstrates that abbreviated food insecurity screeners, including two-item and short-form measures derived from USDA modules, exhibit high sensitivity and specificity relative to more comprehensive instruments [26,27] and produce prevalence estimates and health associations that are broadly comparable, particularly in population-based studies of U.S. adults and older populations [7,9,16,27].

*SNAP participation*. SNAP enrollment was determined by the question to household respondents, “Did you (or other family members who were living here) receive government food stamps at any time since the previous interview/in the last two years?” A response of “yes” to this question was considered as having received SNAP [7,8].

*Covariates*. These included time-invariant covariates and time-varying covariates. Time-invariant covariates included gender, race/ethnicity (non-Hispanic White, non-Hispanic Black, Hispanic, other), education in years, childhood socioeconomic status (mother having 8 or more years of education, father having 8 or more years of education, and whether the family was financially poor), childhood health (self-rating of health in childhood on a 5-point scale ranging from poor to excellent), and HRS cohort status (AHEAD, HRS/AHEAD overlap, CODA, HRS, War babies, early baby boomers, mid baby boomers, and late baby boomers). Time-varying covariates, including age in years, residence (urban, suburban, rural), marital status (married/partnered, divorced/separated, widowed, never married), living alone, employment status (employed, unemployed, retired, not in labor force), annual household income, total household net worth, having health insurance coverage, out-of-pocket medical spending, chronic conditions, Body Mass Index (BMI), depressive symptoms, current smoking, drinking alcohol at least one day per week, vigorous physical activity participation. Household income and medical spending were log-transformed, and total household net worth was converted by the log-modulus transformation [28]. Health insurance coverage was dummy coded, indicating whether respondents were covered by any of the four types of health insurance: Medicare, Medicaid, private health insurance, or military health insurance (yes = 1). The number of chronic conditions was a count of the medical conditions the respondent had ever been diagnosed with, including high blood pressure, diabetes, cancer, lung disease, heart disease, stroke, psychiatric problems, and arthritis. Depressive symptoms were assessed using the short form of the Center for Epidemiological Studies Depression Scale (CESD) by summing up six negative and two positive indicators of mood, with higher scores indicating more severe depressive symptoms.

### 2.3. Statistical Analysis

Descriptive statistics at the baseline survey were calculated for the whole sample and for the three food insecurity groups. ANOVA tests were used for continuous variables, and chi-square tests were used for categorical variables to determine statistical significance of the group differences. We then used two-stage marginal structural models (MSMs) with inverse probability of treatment weighting (IPTW) to estimate the population-averaged effect of food insecurity on cognitive function over time. MSMs are well-suited for longitudinal analyses where time-varying confounders, such as changes in socioeconomic status, health conditions, or prior cognitive function, both influence future exposure to food insecurity and are themselves influenced by past exposure [29,30]. Traditional regression adjustment can yield biased estimates under these conditions, whereas MSMs account for these feedback relationships by creating a weighted sample in which the time-varying confounders are no longer associated with subsequent exposure.

In the first stage, we estimated multinomial IPTWs for each wave because the exposure (i.e., food insecurity) had three categories [30]. For each wave, we fit two multinomial logistic models: the numerator model estimated the probability of each food insecurity category based on time-invariant covariates and prior food insecurity to stabilize the weights and improve precision, and the denominator model estimated the probability of each food insecurity category based on prior food insecurity, lagged cognitive function, time-varying socioeconomic and health covariates, and survey wave. The stabilized weight was calculated as the ratio of the numerator to the denominator probability and the final IPTW at time T was defined as the cumulative product of the stabilized IPTWs across all waves from baseline through time T, yielding a single longitudinal weight per person at each time point [29]. We truncated extreme weights at the 1st and 99th percentiles to reduce the influence of outliers [30]. The IPTWs across all time points had a mean of 0.99 and standard deviation of 0.22 for all three cognition measures.

In the second stage, we estimated the effect of food insecurity on cognitive function, and the modifying role of SNAP participation, using weighted pooled Ordinary Least Squares (OLS) regression models. The first model included food insecurity, survey wave, and time-invariant covariates. The second model added SNAP participation and the interaction term between food insecurity and SNAP participation. Stabilized IPTWs were applied as probability weights, and standard errors were clustered at the respondent level to account for repeated observations [30]. This MSM approach provides marginal (population-level) estimates of the longitudinal association between food insecurity and cognitive performance while appropriately adjusting for time-varying confounding.

We estimated MSMs for overall cognitive function as well as for two cognitive subdomains: episodic memory and attention/mental processing. To assess whether the associations among food insecurity, SNAP participation, and cognitive function differed by race/ethnicity, we repeated the analyses separately for non-Hispanic White, non-Hispanic Black, and Hispanic participants. We did not conduct separate analyses for respondents classified as “other” race due to small sample size. In addition, we conducted stratified MSMs by sex and age group (50–64 vs. ≥65 years) to examine whether associations differed across vulnerable subpopulations.

To aid interpretation of effect sizes, we quantified the clinical magnitude of food insecurity effects on cognitive function using two complementary approaches. First, unstandardized regression coefficients were expressed as standard deviation (SD) units based on the distribution of each cognitive measure at baseline. Second, we derived approximate age-equivalent differences by estimating auxiliary linear regression models relating cognitive scores to age and survey wave, additionally adjusting for sex, race/ethnicity, education, and study cohort. The resulting age coefficients were used solely to contextualize the magnitude of observed associations in terms of years of age-related difference and were not intended to represent causal estimates of cognitive aging. All analyses were conducted using Stata version 18 (StataCorp, College Station, TX, USA).

## 3. Results

### 3.1. Descriptive Statistics

Descriptive statistics for the baseline sample are presented in Table 1. Among all respondents included in the study, approximately 6% reported moderate food insecurity and 6% reported high food insecurity. The average score was 15.77 for overall cognition, 10.35 for episodic memory, and 5.42 for attention/mental processing. About 9% of respondents received SNAP in the previous years. Differences across levels of food insecurity were statistically significant for all variables at the *p* < 0.001 level. Across all three cognitive measures, low food-insecure respondents had the highest mean scores, whereas those experiencing high food insecurity had the lowest scores. The prevalence of SNAP participation was greatest among respondents with high food insecurity and lowest among those with low food insecurity.

Compared with low food-insecure respondents, those with high food insecurity tended to be younger, more likely to be female and members of racial or ethnic minority groups, and less likely to be married or cohabiting. They also had lower levels of educational attainment, were less likely to be employed or retired, and were less likely to have health insurance coverage. In addition, they reported substantially lower household income, household net worth, and medical expenditures. Respondents with high food insecurity also experienced a greater number of chronic health conditions and depressive symptoms. Furthermore, they had higher BMI, were more likely to smoke, but less likely to consume alcohol or engage in vigorous physical activity. For most of these characteristics, those with moderate food insecurity fell between low and high food insecurity groups.

### 3.2. Relationships Between Food Insecurity, SNAP, and Cognitive Function

**Figure 1:**
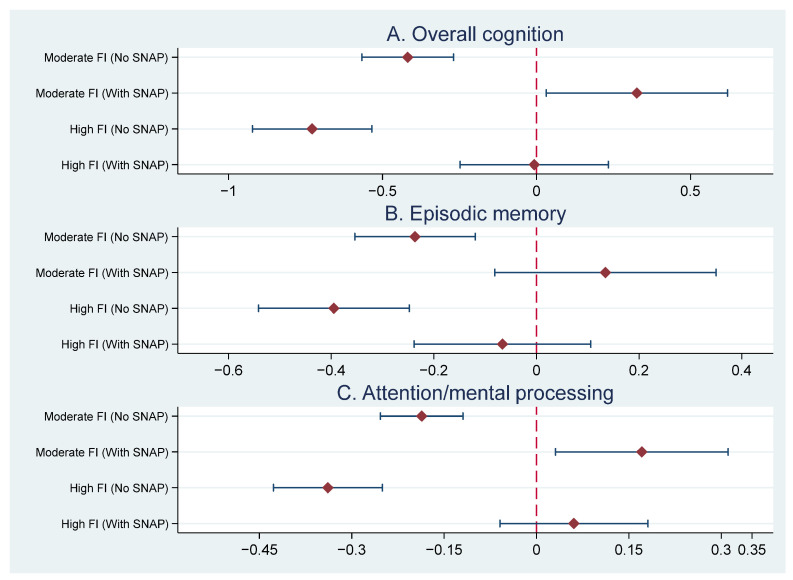
Marginal effects of moderate and high food insecurity on cognitive outcomes by SNAP status, estimated using marginal structural models. Points represent unstandardized regression coefficients and bars indicate 95% confidence intervals. Estimates for SNAP participants reflect the combined main and interaction effects. Panels display associations for three cognitive outcomes measured on different scales; therefore, effect sizes are not directly comparable across panels. FI: Food insecure.

Table 2 presents the results from the MSMs examining the association between food insecurity status and cognitive outcomes for all respondents. A forest plot summarizes key estimates across cognitive domains to facilitate comparison of effect sizes and confidence intervals (Figure 1). After accounting for time-varying confounders through IPTWs and controlling for time-invariant covariates in Model 1, respondents experiencing moderate food insecurity scored 0.36-point lower (95% CI = [−0.50, −0.23], *p* < 0.001) on overall cognition score than low food-insecure respondents, corresponding to 0.08 SD and an estimated 2.4 years of age-related difference. Those with high food insecurity scored 0.71-point lower (95% CI = [−0.87, −0.55], *p* < 0.001), equivalent to 0.16 SD and approximately 4.8 years of age-related difference.

When SNAP participation and the interaction terms between food insecurity categories and SNAP participation were added in Model 2, while the main effects of food insecurity remained negative and significant at *p* < 0.001 level, both interaction terms for moderate food insecurity (interaction b = 0.74, 95% CI = [0.42, 1.07], *p* < 0.001) and for high food insecurity (interaction b = 0.72, 95% CI = [0.43, 1.01], *p* < 0.001) were positive and significant, suggesting that SNAP participation buffered the negative association between food insecurity and cognitive function. Only for respondents who were not participating in SNAP, higher levels of food insecurity were associated with worse overall cognition scores. For SNAP participants, those with moderate food insecurity scored higher on overall cognition (b = 0.74–0.42 = 0.32, *p* < 0.05) and those with high food insecurity were not significantly different than those with low food insecurity.

Table 2 also presents results for the two cognitive subdomains: episodic memory and attention/mental processing. For both domains, food insecurity had a graded negative association with cognitive performance; respondents with moderate and high food insecurity scored lower on both episodic memory and attention/mental processing than low food insecurity respondents, with the greatest deficits observed among those with high food insecurity. Moderate and high food insecurity were associated with 0.22-point (95% CI = [−0.33, −0.12] *p* < 0.001) (0.06 SD) and 0.43-point (95% CI = [−0.55, −0.31], *p* < 0.001) (0.12 SD) lower memory scores, respectively, corresponding to approximately 1.7 and 3.4 years of age-related difference. Moderate and high food insecurity were associated with 0.15-point (95% CI = [−0.21, −0.09], *p* < 0.001) (0.08 SD) and 0.28-point (95% CI = [−0.36, −0.21], *p* < 0.001) (0.16 SD) lower attention/mental processing scores. Because age-related change in this domain was relatively shallow, translating these differences into years yielded larger values and should therefore be interpreted cautiously.

When interaction terms between food insecurity and SNAP participation were added, both interaction terms between food insecurity status and SNAP were statistically significant for both episodic memory and attention/mental processing, indicating that SNAP participation moderated the negative association between food insecurity and both subdomains of cognition. Only for those who were not SNAP participants, food insecurity had a significant negative association with both episodic memory and attention/mental processing. For SNAP participants, food insecurity was not significantly associated with episodic memory. Also, for SNAP participants, moderate food-insecure respondents had higher scores on attention/mental processing than low food-insecure respondents (b = 0.17, *p* < 0.05), while high food-insecure respondents were not significantly different from low food-insecure respondents.

### 3.3. Racial/Ethnic Differences in the Relationship Between Food Insecurity, SNAP, and Cognitive Function

Table 3 presents race/ethnicity-stratified MSM estimates of associations between food insecurity, SNAP participation, and cognitive function. Because interaction terms between food insecurity and SNAP were included, the main effects represent the association between food insecurity and cognition among individuals who were not participating in SNAP. Across all racial/ethnic groups, moderate and high food insecurity were consistently associated with poorer overall cognition among non-SNAP participants. For non-Hispanic Whites, moderate food insecurity was associated with 0.44-point lower cognitive score (95% CI = [−0.63, −0.25], *p* < 0.001), and high food insecurity with 0.97-point lower score (95% CI = [−1.25, −0.69], *p* < 0.001), relative to low food insecurity. Similar patterns were observed among non-Hispanic Blacks (moderate: b = −0.32, 95% CI = [−0.593, −0.049], *p* < 0.05; high: b = −0.31, 95% CI = [−0.624, 0.009], *p* < 0.1) and Hispanics (moderate: b = −0.40, 95% CI = [−0.785, −0.022], *p* < 0.05; high: b = −0.79, 95% CI = [−1.269, −0.306], *p* < 0.01).

SNAP participation itself was associated with lower overall cognition in all groups (b ranges from −0.98 to −1.16). However, significant interaction terms indicate that the association between food insecurity and cognition differed for SNAP participants compared with non-participants. For example, among non-Hispanic Blacks, SNAP participation substantially reduced the cognitive disadvantage linked to moderate food insecurity (interaction b = 0.86, 95% CI = [0.39, 1.34], *p* < 0.001) and high food insecurity (interaction b = 0.61, 95% CI = [0.15, 1.07], *p* < 0.01). Similar moderating effects were observed among Hispanics for both moderate and high food insecurity groups and among non-Hispanic Whites for high food insecurity group.

Patterns were similar across cognitive domains. In episodic memory, food insecurity was associated with significantly lower memory scores among non-SNAP participants for non-Hispanic Whites and Hispanics, and for moderate insecurity among non-Hispanic Blacks. SNAP again moderated these associations, particularly for non-Hispanic Blacks. For attention/mental processing, moderate and high food insecurity were associated with lower scores among non-SNAP non-Hispanic Whites and Hispanics, and high food insecurity was negatively associated with lower scores among non-Hispanic Blacks. SNAP participation moderated these associations: interaction terms for high food insecurity and SNAP were significant and positive in all three racial/ethnic groups, indicating that SNAP participation buffered the negative association between high food insecurity and attention/mental processing.

Stratified analyses by sex and age group showed generally consistent patterns across subgroups, with food insecurity associated with lower cognitive scores, and evidence of SNAP-related buffering in both men and women and in middle-aged and older adults (Supplementary Tables S1 and S2).

## 4. Discussion

This study provides new evidence on the longitudinal relationship between food insecurity, participation in SNAP, and cognitive functioning among U.S. adults aged 50 years and older, using 22 years of nationally representative data. By employing MSMs with IPTWs, we addressed the substantial methodological challenge of time-varying confounding, particularly socioeconomic and health factors that both shape exposure to food insecurity and are influenced by it over time. Three major findings emerged. First, food insecurity was consistently associated with poorer overall cognition, episodic memory, and attention/mental processing. The magnitude of these associations suggests that food insecurity is not only statistically significant but also clinically meaningful in the context of cognitive aging. Second, SNAP participation attenuated or fully eliminated the negative association between food insecurity and cognitive outcomes, such that the adverse relationship was observed only among individuals not receiving SNAP. Third, stratified analyses revealed that these patterns were largely consistent across racial and ethnic groups, although moderation effects appeared especially pronounced among non-Hispanic Black and Hispanic older adults. Together, these findings suggest that food insecurity is an important and potentially modifiable social determinant of cognitive aging, and that SNAP may play a protective role in preserving cognitive health among socioeconomically vulnerable older adults.

### 4.1. Food Insecurity and Cognitive Function in Later Life

Consistent with previous cross-sectional and longitudinal research, higher levels of food insecurity were associated with lower cognitive performance [14,16,17,19,31]. This study extends prior work by demonstrating a graded dose–response pattern across multiple cognitive domains using methodological tools specifically designed to address reverse causation and feedback loops. Older adults experiencing moderate food insecurity had worse cognitive scores than those with low food insecurity, and those with high food insecurity exhibited the greatest deficits. These findings are consistent with conceptual frameworks positing that food insecurity represents an accumulation of material deprivation, nutritional inadequacy, psychological stress, and physiological burden, all of which are well-established determinants of cognitive decline [10,12,21,32].

Although the absolute differences in cognitive scores associated with food insecurity were modest, their magnitude is clinically meaningful when considered relative to population variability and normative age-related change. The observed associations corresponded to differences of approximately 0.06–0.16 SD across cognitive domains and to several years of age-related difference in overall cognition and episodic memory. These magnitudes are comparable to those reported for other social and behavioral risk factors in aging research [33,34] and may accumulate over time to meaningfully influence cognitive trajectories at the population level. Importantly, the cognitive domains most strongly associated with food insecurity, such as overall cognition and episodic memory, are known to be particularly sensitive to psychosocial stressors, cardiometabolic risk, and nutritional inadequacy. By contrast, attention and mental processing tasks exhibited smaller age gradients, consistent with prior evidence that these measures reflect more stable cognitive functions. As a result, age-equivalent translations for this domain should be viewed as heuristic benchmarks rather than direct indicators of accelerated cognitive aging.

The cognitive disadvantages associated with food insecurity may operate through multiple, interrelated nutritional, biological, and psychosocial pathways. Food insecurity is closely linked to poorer dietary quality, including lower consumption of fruits, vegetables, and nutrient-dense foods and higher intake of calorie-dense, nutrient-poor options [35]. Such dietary patterns are associated with deficiencies in nutrients essential for neurocognitive functioning, including B vitamins, omega-3 fatty acids, and antioxidants, which have been implicated in synaptic integrity, neuroinflammation, and cognitive aging processes [36]. In parallel, food insecurity represents a chronic stressor that contributes to elevated allostatic load, increases systemic inflammation, and dysregulation of the hypothalamic–pituitary–adrenal axis [6,7,37]. These stress-related biological responses are particularly relevant for hippocampal structure and function, offering a plausible explanation for the observed associations with episodic memory. Additionally, food insecurity often co-occurs with broader material hardship, including housing instability and constrained healthcare access, which may impair disease management, medication adherence, and opportunities for cognitively stimulating activities, thereby affecting attention and executive functioning [38]. The present findings are consistent with these mechanisms, demonstrating associations across both episodic memory and attention/mental processing domains.

### 4.2. SNAP Participation as a Protective Factor for Cognitive Health

A key contribution of this study is the robust evidence that SNAP participation buffers the negative association between food insecurity and cognitive performance. In every model, including overall cognition, episodic memory, and attention/mental processing, interaction terms indicated that food insecurity predicted lower cognitive function only among individuals not enrolled in SNAP. Among SNAP participants, moderate or high food insecurity was not associated with cognitive disadvantage after accounting for time-varying socioeconomic and health factors. These findings extend earlier work showing that SNAP improves dietary quality [39], reduces psychological distress [40], and improves cardiometabolic risk factors [41]. Our findings are consistent with findings from previous studies [13,15]. Yet, to our knowledge, this is one of the first longitudinal studies using MSMs to show that SNAP may play a role in protecting cognitive health among older adults experiencing food insecurity.

Several interrelated mechanisms may explain the protective effect of SNAP observed in this study. By increasing household food purchasing power, SNAP improves access to more nutritious foods and reduces reliance on lower-quality, energy-dense diets, which may be particularly consequential for episodic memory given its sensitivity to nutritional deficiencies, oxidative stress, and chronic inflammation [36]. At the same time, SNAP participation may alleviate financial strain by freeing household resources for medical care, transportation, and other essential needs, thereby reducing psychological stress and allostatic load, which have been linked to cognitive aging. In addition, engagement with SNAP may enhance social connectedness and access to information about community resources through interactions with social service systems, particularly among older adults. Although these pathways cannot be directly tested in the present study, the consistent moderating patterns across cognitive domains suggest that SNAP’s protective role likely extends beyond nutrition alone, contributing to broader physiological and psychosocial resilience.

### 4.3. Racial/Ethnic Variation in the Associations

Race/ethnicity-stratified analyses provided additional insight into population differences in the relationship among food insecurity, SNAP, and cognition. Although the adverse association between food insecurity and cognition was consistently evident among non-SNAP participants across non-Hispanic White, non-Hispanic Black, and Hispanic groups, moderation by SNAP appeared especially salient for non-Hispanic Black and Hispanic older adults. Among these groups, SNAP substantially reduced or eliminated the cognitive disadvantage linked to moderate and high food insecurity. These findings align with previous literature showing that food insecurity disproportionately affects non-Hispanic Black and Hispanic households [2], and that SNAP participation plays a vital role in reducing nutritional disparities and mitigating financial hardship among racial/ethnic minority populations [42]. Older Black and Hispanic adults face cumulative exposure to socioeconomic disadvantage, structural racism, and chronic stress across the life course, which magnifies vulnerability to cognitive decline [43]. The strong moderating effect of SNAP in these groups may reflect its ability to offset more severe or chronic forms of hardship.

At the same time, SNAP participation was associated with lower overall cognitive function in all racial and ethnic groups, reflecting selection into the program rather than adverse program effects. Individuals participating in SNAP had lower socioeconomic status, poorer baseline health, and higher psychological distress, all of which are strong predictors of lower cognitive performance. The MSM approach adjusts for many of these time-varying characteristics, but it cannot fully eliminate selection effects. The interaction patterns, rather than the main effect of SNAP, therefore, provide more meaningful insight into the program’s impact.

### 4.4. Strengths and Limitations

Several strengths enhance the robustness of this study. The analysis leveraged more than two decades of nationally representative data from the HRS, capturing long-term exposure to food insecurity and repeated cognitive assessments. The use of MSMs with IPTW allowed us to properly address time-varying confounding, which is essential in understanding the interplay between food insecurity, socioeconomic conditions, health status, and cognitive trajectories. Including episodic memory and attention/mental processing as distinct domains enabled domain-specific inference and revealed consistent patterns across both memory and executive functioning. Stratified analyses offered important perspectives on racial and ethnic disparities in food insecurity and its cognitive consequences. Finally, the consistency of findings across subgroups further strengthens confidence in the robustness of the observed associations.

Despite these strengths, several limitations should be acknowledged. First, food insecurity was measured using two household-level items and may not fully capture the multidimensional nature of food access or individual-level experience. Additionally, repeated cognitive testing may introduce practice effects that could influence results, even though survey wave was included to partially address learning-related changes. Second, cognitive assessments were primarily based on the Telephone Interview for Cognitive Status, which, while validated, may not capture finer-grained cognitive deficits. Third, although MSMs address many forms of confounding, unobserved confounding cannot be completely ruled out, and measurement error remains possible. Fourth, information on SNAP benefit amounts or duration of participation was not assessed. Fifth, some observed racial/ethnic differences may reflect unmeasured cultural, social, or structural factors that influence food security, coping strategies, or cognitive aging pathways, which warrant further investigation. More importantly, while many associations were statistically robust, their absolute magnitudes were modest. We therefore interpret these findings as indicators of population-level risk rather than deterministic predictors of individual cognitive decline.

### 4.5. Practical Implications and Future Research Directions

Although the observed effect sizes were modest, the magnitude of cognitive differences associated with food insecurity, equivalent to several years of age-related cognitive change, suggests potential relevance for routine aging care. These findings support the integration of brief food insecurity screening into clinical encounters with middle-aged and older adults, particularly in primary care, geriatric, and nutrition-focused settings [44]. Clinicians, including physicians, dietitians, and social workers, are well positioned to identify food-insecure patients and facilitate referrals to nutrition assistance programs such as SNAP. The attenuated associations observed among SNAP participants suggest that nutrition assistance may help buffer cognitive vulnerability linked to food insecurity, reinforcing the role of nutrition support as a component of cognitive health promotion [45]. Integrating food security assessment into cognitive risk evaluations may therefore enhance early identification and intervention efforts in aging populations.

At the population level, these findings highlight the potential cognitive relevance of nutrition assistance policies. The consistent attenuation of food insecurity and cognition associations among SNAP participants suggests that food assistance programs may contribute to healthier cognitive aging, particularly among socioeconomically disadvantaged groups. Policy efforts aimed at improving SNAP accessibility, benefit adequacy, and continuity of enrollment, especially among older adults and racial/ethnic minority populations, may yield cognitive health benefits. Framing food assistance as an investment in healthy aging may further strengthen cross-sector efforts linking nutrition policy, aging services, and cognitive health promotion [46].

Future studies should examine whether specific dimensions of food insecurity, such as chronicity, dietary quality, or nutritional adequacy, are differentially associated with cognitive domains. Incorporating biomarkers, dietary intake data, or neuroimaging could help clarify underlying mechanisms linking food insecurity to cognitive aging [47]. Quasi-experimental studies evaluating changes in SNAP eligibility or benefit levels may provide stronger causal evidence regarding the cognitive effects of nutrition assistance across the life course [48].

## 5. Conclusions

This study provides longitudinal evidence that food insecurity is associated with poorer cognitive function across multiple domains in later life and that SNAP participation may partially buffer these associations. Although the observed differences in cognitive scores were modest, their magnitude was meaningful when considered relative to population variability and age-related cognitive change, underscoring the potential public health relevance of food insecurity as a modifiable social and nutritional risk factor. The findings highlight the critical role of SNAP as a protective resource for older adults and support public health and policy efforts of improving access to food assistance programs, reducing barriers to SNAP enrollment, and ensuring adequate benefit levels, particularly for socioeconomically disadvantaged and racial/ethnic minority older adults. Future research should examine how benefit levels, dietary quality, chronic stress, and social context jointly shape cognitive trajectories across the later life course using designs that further strengthen causal inference.

## Figures and Tables

**Table 1:** Descriptive statistics at baseline.

	All (N = 30,641)	Low Food Insecurity (N = 27,063)	Moderate Food Insecurity (N = 1772)	High Food Insecurity (N = 1806)
Overall cognition (0–27)	15.77 (4.42)	16.00 (4.35)	14.17 (4.67)	13.78 (4.45)
Episodic memory (0–20)	10.35 (3.49)	10.48 (3.48)	9.41 (3.50)	9.21 (3.28)
Attention/mental processing (0–7)	5.42 (1.79)	5.52 (1.73)	4.76 (2.03)	4.57 (2.02)
SNAP status	9.44	6.23	24.04	43.19
Age	60.96 (9.59)	61.30 (9.70)	60.77 (9.30)	56.08 (6.18)
Female	57.42	56.82	61.12	62.79
Race/ethnicity				
Non-Hispanic white	65.65	69.59	34.99	36.60
Non-Hispanic black	18.58	16.83	24.83	38.65
Hispanic	12.31	10.26	36.74	19.05
Other	3.46	3.31	3.44	5.70
Residence				
Urban	53.01	53.01	52.26	53.77
Sub-urban	20.84	20.32	26.30	23.26
Rural	26.15	26.67	21.44	22.98
Mother’s education ≥ 8 years	68.55	70.44	48.19	60.30
Father’s education ≥ 8 years	59.46	61.55	41.08	46.23
Family poor at age 16	30.23	28.95	39.33	40.48
Childhood health	4.18 (1.00)	4.22 (0.98)	4.00 (1.07)	3.87 (1.19)
Education	12.52 (3.24)	12.72 (3.07)	10.40 (4.44)	11.54 (3.25)
Marital status				
Married/partnered	68.36	70.38	60.16	46.07
Divorced/separated	14.35	12.86	19.47	31.62
Widowed	12.05	12.02	14.11	10.41
Never married	5.24	4.73	6.26	11.90
Living alone	18.51	17.90	19.07	27.13
Employment status				
Employed	46.11	47.10	39.45	37.87
Unemployed	3.28	2.82	4.40	8.97
Retired	37.94	38.65	35.78	29.40
Not in labor force	12.67	11.42	20.37	23.75
Household income (ln)	10.39 (1.63)	10.51 (1.52)	9.58 (2.12)	9.37 (2.01)
Total net worth (log-modulus)	9.69 (5.87)	10.33 (5.17)	6.86 (7.12)	2.74 (8.41)
Health insurance	88.98	90.53	79.51	75.08
Medical expenditure (ln)	5.92 (2.82)	6.04 (2.71)	5.12 (3.31)	4.99 (3.57)
Chronic conditions	1.47 (1.30)	1.42 (1.26)	1.62 (1.42)	2.13 (1.58)
BMI	28.21 (5.93)	28.00 (5.72)	29.04 (6.47)	30.52 (7.65)
Depressive symptoms	1.62 (2.02)	1.46 (1.89)	2.35 (2.32)	3.28 (2.56)
Current smoking	19.39	17.81	22.23	40.20
Drinking alcohol at least 1 day/week	37.02	38.00	28.16	31.06
Vigorous exercise	37.95	39.56	30.7	20.99

Notes: Numbers are mean (standard deviation) or percentage.

**Table 2:** Associations between food insecurity, SNAP, and overall cognition, episodic memory, and attention/mental processing from marginal structural models.

	Model 1		Model 2	
	b	95% CI	b	95% CI
**Overall cognition**				
Food insecurity (ref = low insecurity)				
Moderate insecurity	−0.364 ***	[−0.500, −0.228]	−0.419 ***	[−0.568, −0.270]
High insecurity	−0.710 ***	[−0.869, −0.550]	−0.729 ***	[−0.923, −0.535]
SNAP			−1.172 ***	[−1.309, −1.034]
Moderate food insecurity × SNAP			0.744 ***	[0.424, 1.065]
High food insecurity × SNAP			0.721 ***	[0.433, 1.010]
**Episodic memory**				
Food insecurity (ref = low insecurity)				
Moderate insecurity	−0.224 ***	[−0.329, −0.119]	−0.237 ***	[−0.354, −0.120]
High insecurity	−0.430 ***	[−0.547, −0.312]	−0.395 ***	[−0.542, −0.248]
SNAP			−0.763 ***	[−0.861, −0.664]
Moderate food insecurity × SNAP			0.371 **	[0.131, 0.612]
High food insecurity × SNAP			0.328 **	[0.112, 0.545]
**Attention/mental processing**				
Food insecurity (ref = low insecurity)				
Moderate insecurity	−0.148 ***	[−0.210, −0.086]	−0.186 ***	[−0.253, −0.119]
High insecurity	−0.284 ***	[−0.360, −0.207]	−0.339 ***	[−0.427, −0.250]
SNAP			−0.415 ***	[−0.478, −0.351]
Moderate food insecurity × SNAP			0.357 ***	[0.206, 0.509]
High food insecurity × SNAP			0.399 ***	[0.264, 0.534]
Respondents	30,641		30,641	
Observations	156,066		156,066	

Notes: Estimates are from pooled OLS regressions that were adjusted for clustering and account for gender, race/ethnicity, age, residence, mother’s education, father’s education, family poor at age 16, childhood health, education, marital status, living alone, employment status, household income, total net worth, health insurance, and medical spending, chronic conditions, obesity, depressive symptoms, smoking, alcohol drinking, and physical activity participation, cohort, and survey year by inverse probability treatment weights. All models control for sex, race/ethnicity, mother’s education, father’s education, childhood family poor, childhood health, respondent’s education, cohort, and survey year. b-unstandardized coefficients; CI-confidence interval. *** *p* < 0.001, ** *p* < 0.01 (two-tailed tests).

**Table 3:** Associations between food insecurity, SNAP status and cognition by race/ethnicity from marginal structural models.

	Non-Hispanic White		Non-Hispanic Black		Hispanic	
	b	95% CI	b	95% CI	b	95% CI
**Overall cognition**						
Food insecurity (ref = low insecurity)						
Moderate insecurity	−0.436 ***	[−0.626, −0.247]	−0.321 *	[−0.593, −0.049]	−0.404 *	[−0.785, −0.022]
High insecurity	−0.968 ***	[−1.250, −0.686]	−0.308	[−0.624, 0.009]	−0.787 **	[−1.269, −0.306]
SNAP	−1.052 ***	[−1.260, −0.843]	−0.981 ***	[−1.213, −0.749]	−1.160 ***	[−1.462, −0.858]
Moderate food insecurity × SNAP	0.283	[−0.262, 0.828]	0.863 ***	[0.389, 1.337]	0.872 *	[0.054, 1.690]
High food insecurity × SNAP	0.513 *	[0.055, 0.970]	0.609 **	[0.148, 1.070]	0.839 *	[0.174, 1.505]
**Episodic memory**						
Food insecurity (ref = low insecurity)						
Moderate insecurity	−0.283 ***	[−0.435, −0.131]	−0.226 *	[−0.437, −0.015]	−0.106	[−0.390, 0.179]
High insecurity	−0.537 ***	[−0.759, −0.315]	−0.128	[−0.372, 0.116]	−0.491 **	[−0.833, −0.149]
SNAP	−0.618 ***	[−0.775, −0.462]	−0.754 ***	[−0.920, −0.587]	−0.774 ***	[−0.974, −0.573]
Moderate food insecurity × SNAP	0.119	[−0.298, 0.536]	0.559 **	[0.195, 0.922]	0.279	[−0.299, 0.856]
High food insecurity × SNAP	0.192	[−0.162, 0.546]	0.305	[−0.044, 0.654]	0.421	[−0.055, 0.897]
**Attention/mental processing**						
Food insecurity (ref = low insecurity)						
Moderate insecurity	−0.153 ***	[−0.235, −0.071]	−0.099	[−0.238, 0.040]	−0.307 ***	[−0.487, −0.128]
High insecurity	−0.433 ***	[−0.555, −0.311]	−0.174 *	[−0.335, −0.014]	−0.315 **	[−0.515, −0.115]
SNAP	−0.446 ***	[−0.540, −0.353]	−0.228 ***	[−0.338, −0.119]	−0.388 ***	[−0.532, −0.244]
Moderate food insecurity × SNAP	0.140	[−0.093, 0.374]	0.301 *	[0.064, 0.538]	0.562 **	[0.209, 0.915]
High food insecurity × SNAP	0.324 **	[0.125, 0.523]	0.308 **	[0.083, 0.534]	0.425 **	[0.113, 0.738]
Respondents	20,115		5692		3773	
Observations	109,932		25,370		16,522	

Notes: Estimates are from pooled OLS regressions adjusted for clustering and weighted by inverse probability treatment weights. All models control for sex, mother’s education, father’s education, childhood family poor, childhood health, respondent’s education, cohort, and survey year. *** *p* < 0.001, ** *p* < 0.01, * *p* < 0.05 (two-tailed tests).

## Data Availability

The data used in this study are publicly available from the Health and Retirement Study (HRS), sponsored by the National Institute on Aging (U01AG009740) and conducted by the University of Michigan. Public-use data files can be accessed at the HRS website upon registration: https://hrs.isr.umich.edu/data-products (accessed on 23 September 2025).

## Supplementary Materials

The following supporting information can be downloaded at: https://www.mdpi.com/article/10.3390/nu18020363/s1, Figure S1: Flow chart of sample selection process; Table S1: Associations between food insecurity, SNAP status and cognition by sex from marginal structural models; Table S2: Associations between food insecurity, SNAP status and cognition by age groups from marginal structural models.

## Abbreviations

The following abbreviations are used in this manuscript:

BMI	Body Mass Index
HFSSM	Household Food Security Survey Module
HRS	Health and Retirement Study
IPTW	Inverse Probability of Treatment Weight
MSM	Marginal Structural Model
OLS	Ordinary Least Squares
SNAP	Supplemental Nutrition Assistance Program
U.S.	United States
USDA	United States Department of Agriculture
