# Supplementary material for: Food Insecurity, Supplemental Nutrition Assistance Program Participation and Cognitive Function Among Middle-Aged and Older Adults: Longitudinal Evidence from the Health and Retirement Study
Source: Nutrients. 2026 Jan 22;18(2):363. doi: 10.3390/nu18020363 (PMC12844725; doi:10.3390/nu18020363)
Supplement: Supplementary file 1 [file nutrients-18-00363-s001.zip › nutrients-4070142-supplementary.pdf]

Food Insecurity, Supplemental Nutrition Assistance Program Participation and Cognitive Function Among Middle-Aged and Older Adults: Longitudinal Evidence from the Health and Retirement Study

Supplementary Materials

Figure S1. Flow chart of sample selection process

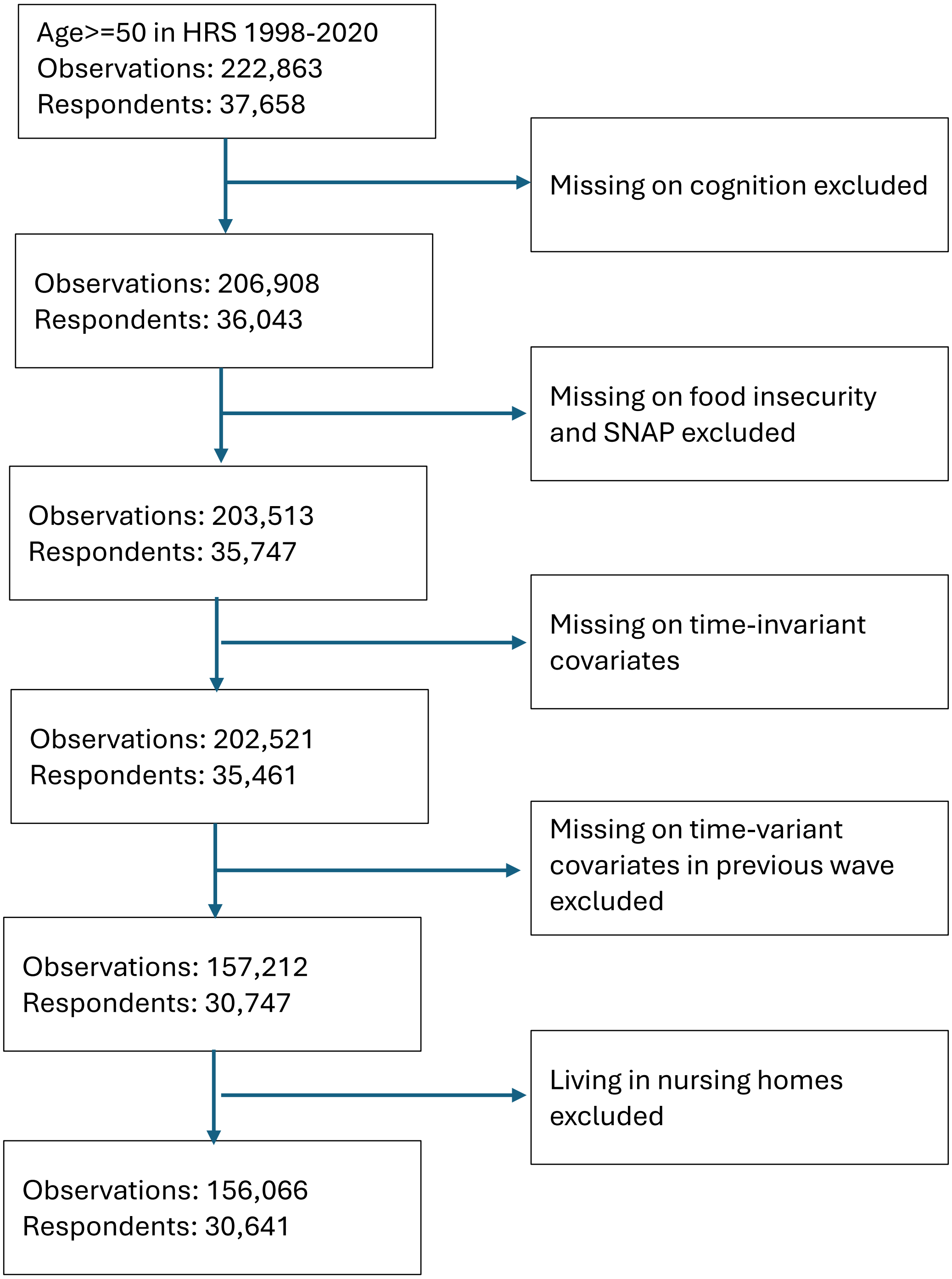

Table S1: Associations between food insecurity, SNAP status and cognition by sex from marginal structural models.

	Men		Women	
	b	95% CI	b	95% CI
**Overall cognition**				
Food insecurity (ref=low insecurity)				
Moderate insecurity	−0.383**	[−0.627, −0.139]	−0.434***	[−0.621, −0.246]
High insecurity	−0.830***	[−1.131, −0.529]	−0.652***	[−0.899, −0.405]
SNAP	−1.125***	[−1.360, −0.889]	−1.147***	[−1.316, −0.979]
Moderate food insecurity × SNAP	0.886**	[0.331, 1.440]	0.702***	[0.313, 1.092]
High food insecurity × SNAP	0.747**	[0.231, 1.263]	0.695***	[0.347, 1.044]
**Episodic memory**				
Food insecurity (ref=low insecurity)				
Moderate insecurity	−0.200*	[−0.385, −0.015]	−0.253**	[−0.404, −0.101]
High insecurity	−0.454***	[−0.680, −0.228]	−0.338***	[−0.526, −0.150]
SNAP	−0.715***	[−0.883, −0.548]	−0.759***	[−0.880, −0.637]
Moderate food insecurity × SNAP	0.400	[−0.011, 0.812]	0.375*	[0.080, 0.670]
High food insecurity × SNAP	0.445*	[0.061, 0.828]	0.283*	[0.020, 0.546]
**Attention/mental processing**				
Food insecurity (ref=low insecurity)				
Moderate insecurity	−0.186**	[−0.298, −0.074]	−0.187***	[−0.270, −0.104]
High insecurity	−0.389***	[−0.531, −0.247]	−0.315***	[−0.427, −0.204]
SNAP	−0.419***	[−0.531, −0.308]	−0.392***	[−0.469. −0.316]
Moderate food insecurity × SNAP	0.479***	[0.206, 0.751]	0.308***	[0.128, 0.488]
High food insecurity × SNAP	0.317**	[0.077, 0.557]	0.415***	[0.252, 0.578]
Respondents	13,046		17,595	
Observations	63,437		92,629	

Notes: Estimates are from pooled OLS regressions adjusted for clustering and weighted by inverse probability treatment weights. All models control for race/ethnicity, mother's education, father's education, childhood family poor, childhood health, respondent's education, cohort, and survey year.

*** $p < 0.001$, ** $p < 0.01$, * $p < 0.05$ (two-tailed tests)

Table S2: Associations between food insecurity, SNAP status and cognition by age groups from marginal structural models.

	Middle-aged adults (50-64)		Older adults (65+)	
	b	95% CI	b	95% CI
**Overall cognition**				
Food insecurity (ref=low insecurity)				
Moderate insecurity	−0.220*	[−0.432, −0.008]	−0.579***	[−0.775, −0.383]
High insecurity	−0.826***	[−1.041, −0.610]	−0.598***	[−0.916, −0.279]
SNAP	−1.468***	[−1.638, −1.298]	−0.951***	[−1.144, −0.759]
Moderate food insecurity × SNAP	0.510*	[0.085, 0.936]	1.057***	[0.575, 1.539]
High food insecurity × SNAP	0.969***	[0.636, 1.302]	0.516*	[0.017, 1.015]
**Episodic memory**				
Food insecurity (ref=low insecurity)				
Moderate insecurity	−0.153	[−0.330, 0.024]	−0.310***	[−0.459, −0.160]
High insecurity	−0.528***	[−0.695, −0.361]	−0.206	[−0.449, 0.037]
SNAP	−1.032***	[−1.156, −0.907]	−0.546***	[−0.682, −0.409]
Moderate food insecurity × SNAP	0.252	[−0.070, 0.574]	0.608**	[0.243, 0.974]
High food insecurity × SNAP	0.615***	[0.359, 0.870]	0.074	[-0.298, 0.446]
**Attention/mental processing**				
Food insecurity (ref=low insecurity)				
Moderate insecurity	−0.069	[−0.157, 0.018]	−0.275***	[−0.368, −0.182]
High insecurity	−0.302***	[−0.398, −0.205]	−0.398***	[−0.542, −0.253]
SNAP	−0.445***	[−0.525, −0.365]	−0.408***	[−0.496, −0.320]
Moderate food insecurity × SNAP	0.245*	[0.056, 0.434]	0.428***	[0.198, 0.658]
High food insecurity × SNAP	0.365***	[0.208, 0.522]	0.440***	[0.206, 0.675]
Respondents	19,859		20,687	
Observations	61,244		94,822	

Notes: Estimates are from pooled OLS regressions adjusted for clustering and weighted by inverse probability treatment weights. All models control for sex, race/ethnicity, mother's education, father's education, childhood family poor, childhood health, respondent's education, cohort, and survey year.
*** $p < 0.001$, ** $p < 0.01$, * $p < 0.05$ (two-tailed tests)
